# Inhibitory efficiency of *Andrographis paniculata* extract on viral multiplication and nitric oxide production

**DOI:** 10.1038/s41598-023-46249-y

**Published:** 2023-11-13

**Authors:** Ittipon Siridechakorn, Parvapan Bhattarakosol, Thanayod Sasivimolrattana, Sasiprapa Anoma, Eakkaluk Wongwad, Nitra Nuengchamnong, Ekasit Kowitdamrong, Siwaporn Boonyasuppayakorn, Neti Waranuch

**Affiliations:** 1 Cosmetics and Natural Products Research Center, Faculty of Pharmaceutical Sciences, Naresuan University, Phitsanulok, 65000 Thailand; 2 Center of Excellence in Applied Medical Virology, Department of Microbiology, Faculty of Medicine, Chulalongkorn University, Pathumwan, Bangkok, 10330 Thailand; 3 Department of Cosmetic Sciences, School of Pharmaceutical Sciences, University of Phayao, Phayao, 56000 Thailand; 4 Faculty of Science, Science Laboratory Centre, Naresuan University, Phitsanulok, 65000 Thailand; 5 Department of Pharmaceutical Technology, Faculty of Pharmaceutical Sciences and Center of Excellence for Innovation in Chemistry, Naresuan University, Phitsanulok, 65000 Thailand

**Keywords:** Biotechnology, Cell biology, Chemical biology

## Abstract

*Andrographis paniculata* (Burm. F.) Nees is a medicinal plant previously reported with broad-spectrum antivirals but the mode of inhibition remains elusive. The objective of this study was to identify the most active fraction from *A. paniculata* ethanol extract (APE, APE-2A, APE-2B and APE-2C) and dry powder extract (APSP) against influenza A (H3N2), representing RNA viruses, and herpes simplex virus-1 (HSV-1), representing DNA viruses. The results showed that the fractions APSP, APE, APE-2B, and APE-2C directly neutralized the HSV-1 and influenza A (H3N2) when incubated at room temperature for 60 min before infecting the cells. The results also showed that the additional APE-2A fraction also directly neutralized the influenza A (H3N2), but not the HSV-1. The APE, APE-2B and APE-2C inhibited the HSV-1 by more than 0.5 log when the fractions were introduced after infection. Similarly, the APSP and APE inhibited the influenza A (H3N2) more than 0.5 log after infection. Only 50 μg/mL APE-2C inhibited the viruses greater than 0.5 log. In addition, A. *paniculata* extracts were also evaluated for their interfering capacities against nitric oxide (NO) production in LPS-activated RAW 264.7 macrophages. As well, APE-2C potently inhibited NO production at the IC_50_ of 6.08 μg/mL. HPLC and LC–MS analysis indicated that the most actively antiviral fractions did not contain any andrographolide derivatives, whereas the andrographolide-rich fractions showed moderate activity.

## Introduction

*Andrographis paniculata* (Burm. F.) Nees, commonly known in Thailand as “Fah Thalai Jone” belongs to the Acanthaceae family and is found throughout tropical and subtropical Asia and Southeast Asia^1^. *A. paniculata* extracts exhibit a wide range of pharmacological activities such as immunostimulatory^1,2^, antiviral^3,4^, and antibacterial activities^5^. *A. paniculata* extracts contain several constituents with a high content of andrographolide. The extract has broad-spectrum antiviral properties including the possibility of in vitro and in vivo anti-HIV^6^, as well as in vitro anti-dengue virus and chikungunya virus activity^7^. *A. paniculata* extracts are effective against the herpes simplex virus type 1 (HSV-1) that causes herpes^8^ as well as reduced the inflammation caused by influenza viruses^9,10^. In addition, these extracts have also been reported to inhibit the division of influenza viruses^11^, hepatitis C virus^12^, and anti-viral mutations that cause resistance to such antiretroviral drugs^13^. The anti-inflammatory^14^ and anti-allergic activities^15^ of *A. paniculata* have been attributed to andrographolide, which is the major active compound^16,17^.

Because of the COVID-19 pandemic that first arose in December 2019, this virus poses a serious risk to patients. The key mechanism for the manifestation of this disease is the inflammation process, which was the focus of this research which investigated the antiviral efficacy and anti-inflammation of *A. paniculata* extract. As SARS-CoV2, a causative agent of COVID-19 is an enveloped RNA virus, influenza virus A (H3N2) was chosen as the representative. In addition, HSV-1 was also selected as an enveloped DNA virus as a surrogate for the COVID-19 virus. The anti-inflammatory activities were tested by a nitric oxide inhibitory assay using the Griess reaction.

## Results

### Cytotoxicity assay

**Figure 1:**
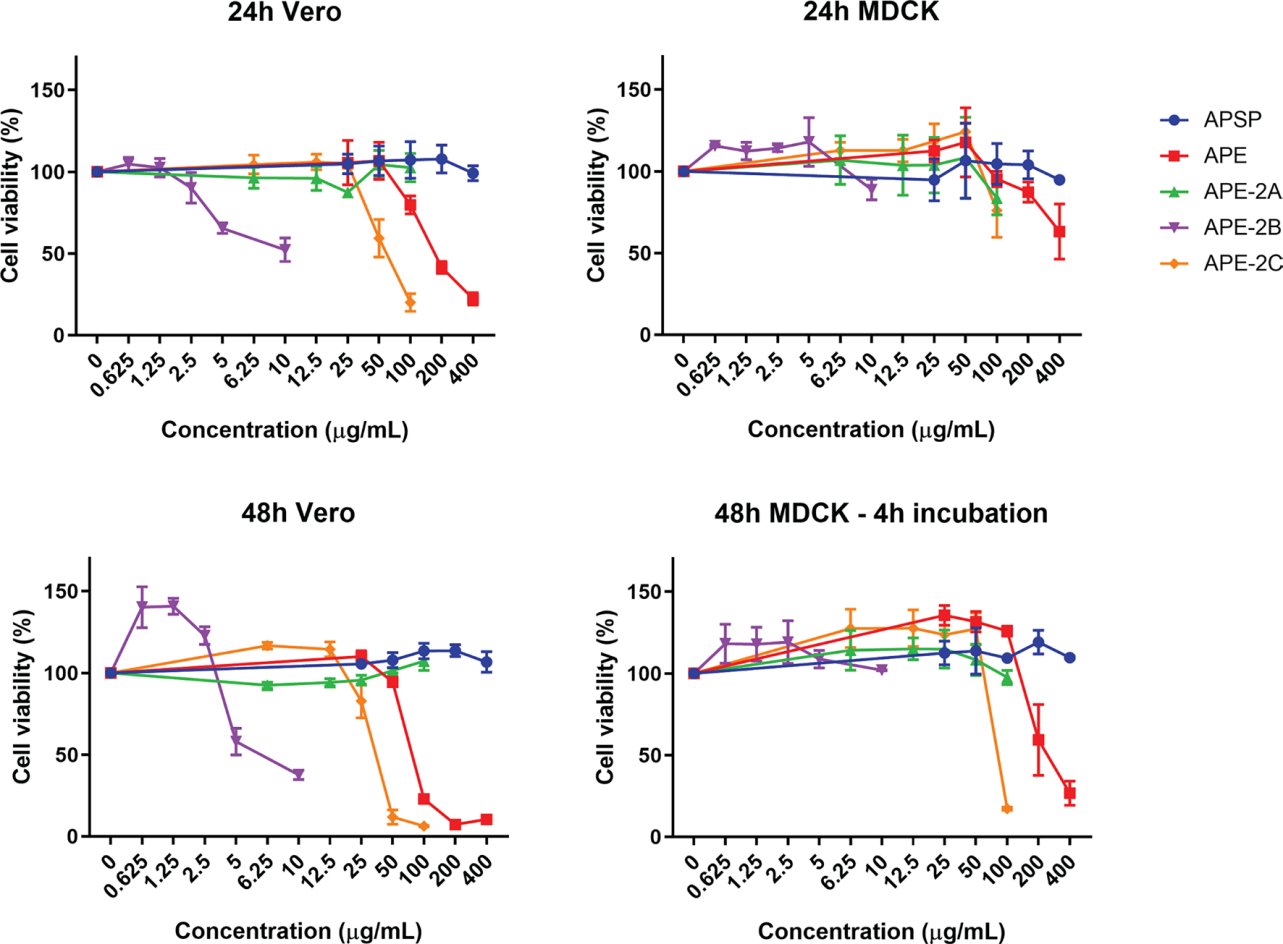
Cytotoxicity assay by MTS for 4 h of Vero and MDCK cells at 24 and 48 h reaction with various concentrations of *A. paniculata* extracts as follows: APSP, APE, APE-2A, APE-2B, and APE-2C.

**Figure 2:**
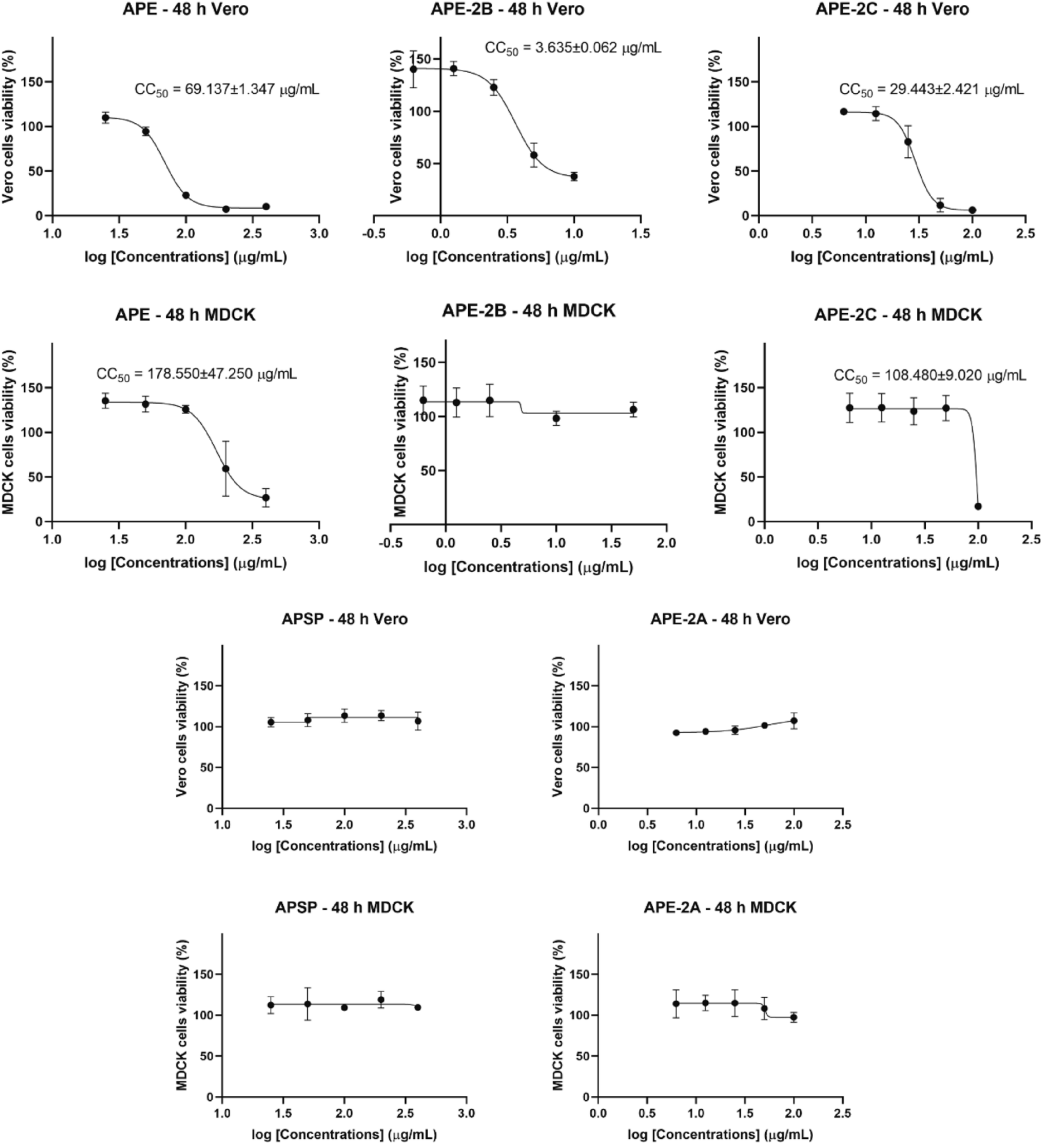
Cytotoxicity concentration (CC_50_) of *A. paniculata* extracts as follows; APSP, APE, APE-2A, APE-2B, and APE-2C in Vero cells and MDCK cells. Data represented Mean ± standard error of mean (SEM) of three independent experiments.

To evaluate the cytotoxicity of *A. paniculata* extract fractions, i.e., APSP, APE, APE-2A, APE-2B and APE-2C, various concentrations were tested against Vero cells and Madin-Darby Canine Kidney (MDCK) cells using the colorimetric MTS assay. After 24 and 48 h of reaction, the living cells (% cell viability) were measured. The results indicated that the cytotoxicity of each extract was different depending on reaction time, cell type and concentration (Figs. 1 and 2). In both Vero and MDCK cells, APSP, and APE-2A had no toxicity in every concentration (Fig. 1). On the contrary, APE, APE-2B and APE-2C showed cytotoxicity at 24 h and increased at 48 h. Interestingly, APE-2B showed cytotoxicity only in Vero cells (Figs. 1 and 2). The 50% cell cytotoxicity were calculated and shown in Fig. 2. Vero cells seemed to be more sensitive to *A. paniculata* extracts (APE, APE-2C and especially APE-2B) than MDCK.

### Direct effect of *A. paniculata* extracts on virus particles (pre-exposure experiment)

**Figure 3:**
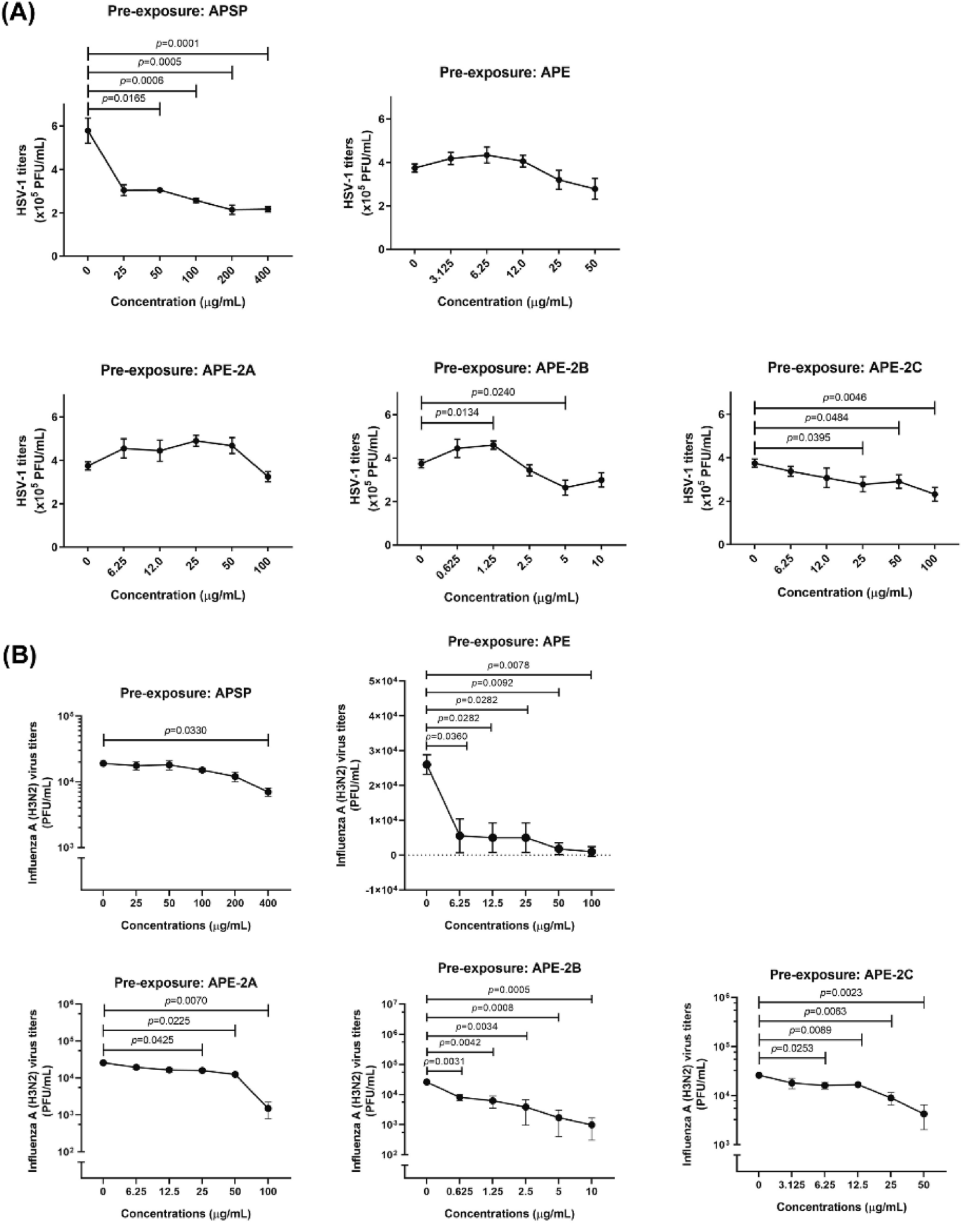
Pre-exposure experiment of *A. paniculata* extracts as follows; APSP, APE, APE-2A, APE-2B, and APE-2C against HSV-1 in Vero cell (**A**) and Influenza A (H3N2) in MDCK cell (**B**). Data represented means ± standard error of mean (SEM) of three independent experiments. *p-value* indicated significant difference between treated and control groups (unpaired t-test).

To observe whether the *A. paniculata* extracts (APSP, APE, APE-2B and APE-2C) could directly inactivate the virus infectivity, pre-exposure experiment was performed. APSP, APE-2B and APE-2C were able to destroy the HSV-1 virus particles directly except for APE-2A, while they all were able to destroy the influenza A (H3N2), viral particles directly (Fig. 3). Viral inhibition concentration (IC_50_) was calculated comparing to the control viruses without the extracts (Table 1). Acyclovir treatment, a common anti-HSV-1 drug, was used as a positive control (Supplemental Fig. 1).

**Table 1:** Inhibition concentration (IC_50_) of pre-exposure experiment against HSV-1 and influenza A (H3N2).

Extract	HSV-1 (IC_50,_ µg/mL)	Influenza A (H3N2) (IC_50,_ µg/mL)
APSP	99.860	275.800
APE	20.200	38.890
APE-2A	NE	UD
APE-2B	2.430	1.837
APE-2C	11.420	22.860

NE, no effect; UD, unable to determine due to the limitation of the compound toxicity.

### The efficiency of *A.paniculata* extracts in inhibiting viral growth (post-exposure experiment)

**Figure 4:**
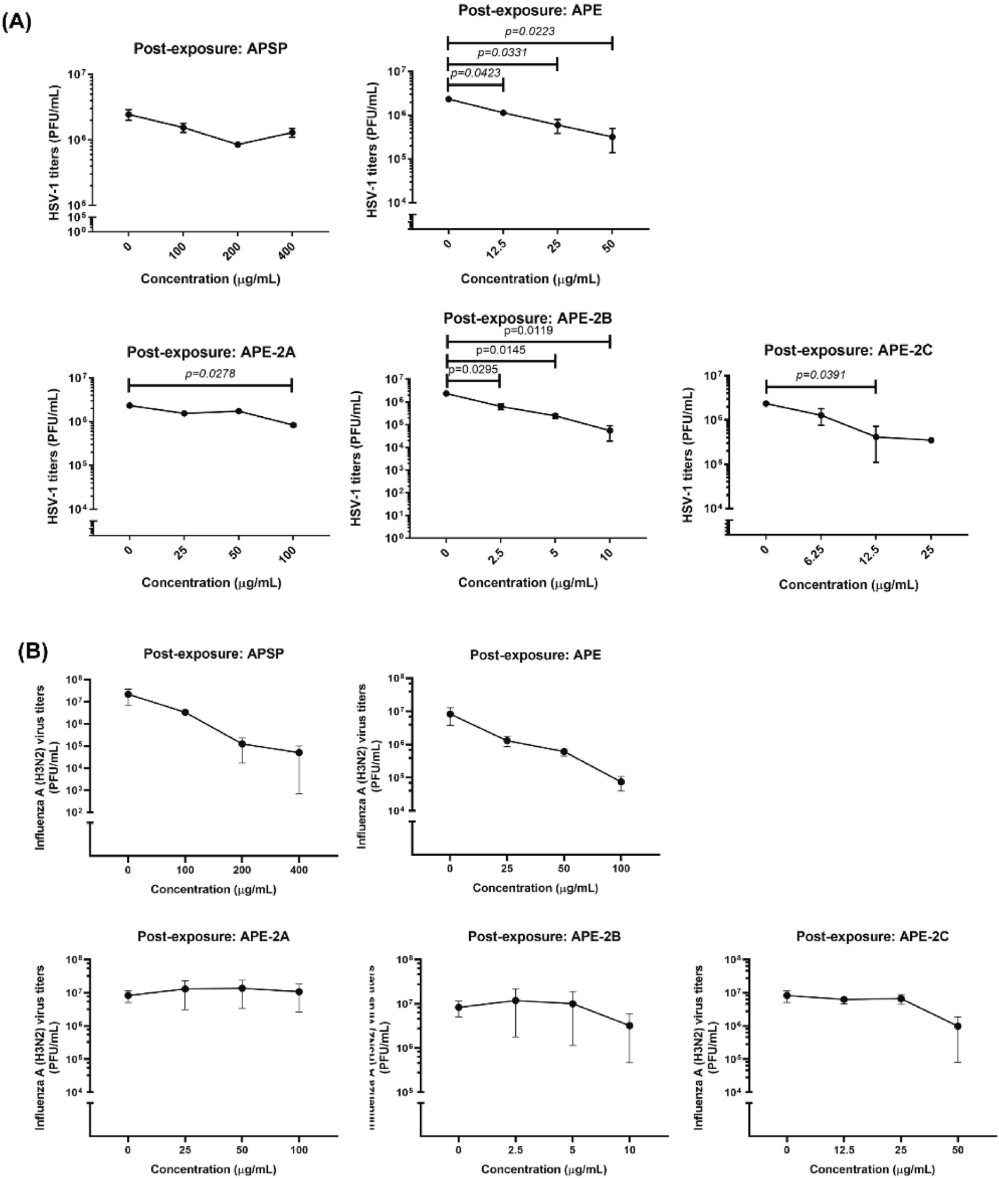
Post-exposure experiment of *A. paniculata* extracts as follows; APSP, APE, APE-2A, APE-2B, and APE-2C against HSV-1 in Vero cell (**A**) and Influenza A (H3N2) in MDCK cell (**B**). Data represented means ± standard error of mean (SEM) of three independent experiments. *p-value* indicated significant difference between treated and control groups (unpaired t-test).

**Figure 5:**
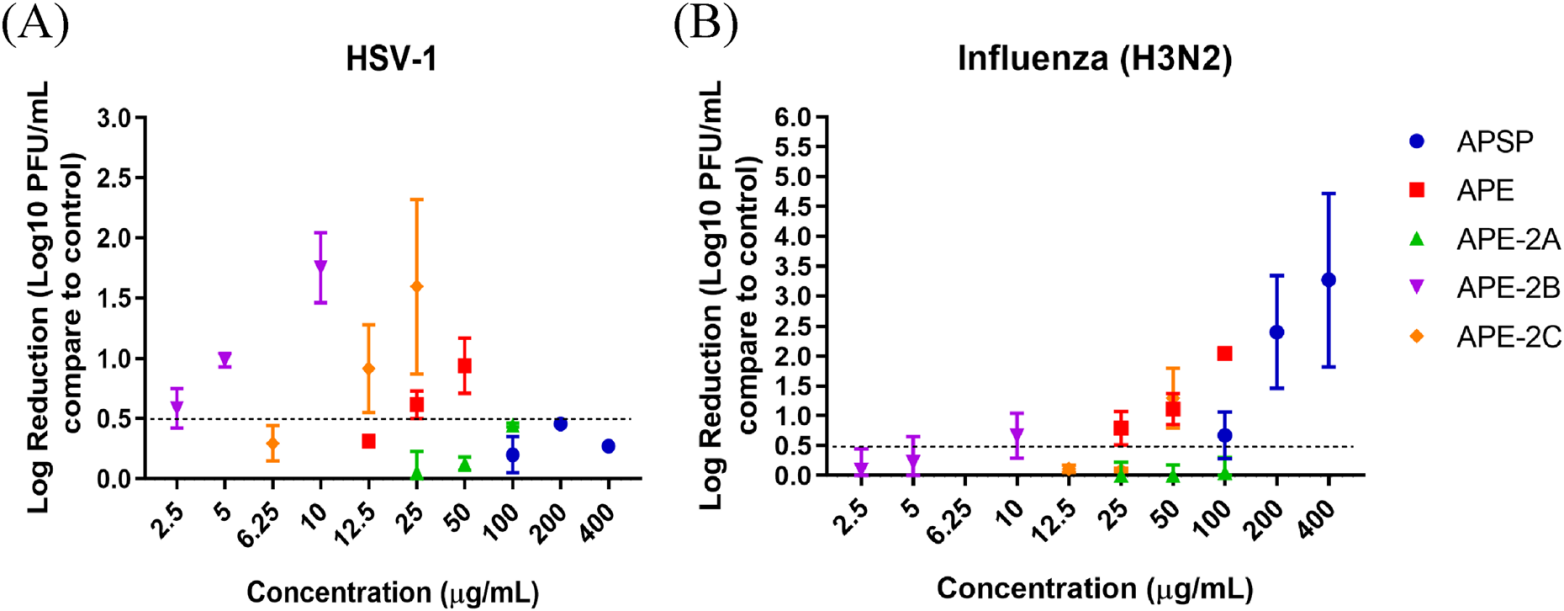
Log10 reduction of HSV-1 (**A**) and Influenza A (H3N2) (**B**) after post-exposure with various concentrations of *A. paniculata* extracts. Error bar represented (SEM).

Whether those 5 fractions of *A. paniculata* extracts could inhibit the viral growth in infected cells, post-exposure experiment was done. APE, APE-2B and APE-2C had ability to inhibit the HSV-1 virus multiplication greater than 0.5 log as well as APSP, APE-2B and APE-2C inhibited the multiplication of influenza A (H3N2) (Figs. 4 and 5). Viral inhibition concentration (IC_50_) was calculated comparing to the control viruses without the extracts (Table 2).

**Table 2:** IC_50_ of post-exposure experiment against HSV-1 and Influenza A (H3N2).

Extract	HSV-1 (IC_50,_ µg/mL)	Influenza A (H3N2) (IC_50,_ µg/mL)
APSP	UD	114.500
APE	14.010	43.640
APE-2A	NE	NE
APE-2B	3.892	7.327
APE-2C	7.692	47.520

NE, no effect; UD, unable to determine due to the limitation of the compound toxicity.

### NO inhibitory activity

**Figure 6:**
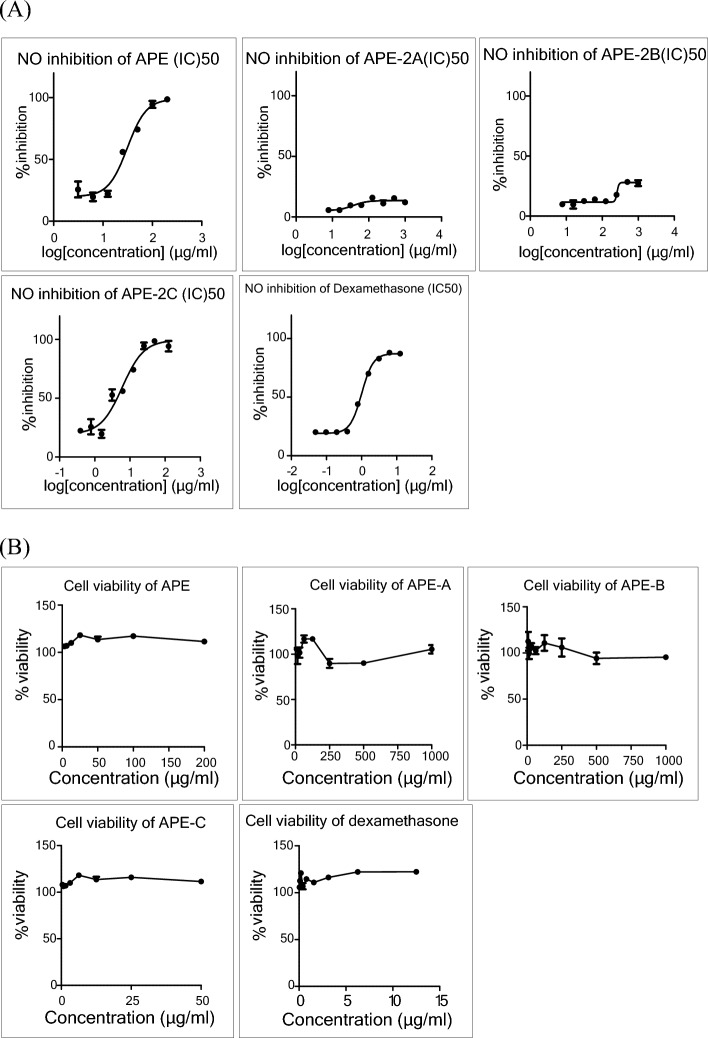
Nitric oxide inhibitory activity (**A**) and cell viability (**B**) at the effective doses.

Nitric oxide released within damaged tissue is an important mediator in the regulation of inflammation^18^. The extracts were evaluated for their inhibiting NO production activity in LPS-activated RAW 264.7 macrophages. Dexamethasone was used as a positive control (IC_50_ = 1.00 μg/mL). The results indicated that APE-2C was the most potent activity fraction (IC_50_ = 6.08 μg/mL) and APE was a moderate activity fraction (IC_50_ = 31.14 μg/mL) as presented in Fig. 6A, Table 3. None of the extracts was toxic the cells, as measured by the MTT assay at the tested concentration, which confirms that the reduction in NO levels is not directly related to cell death (Fig. 6B).

**Table 3:** NO inhibitory effects of *A. paniculata* extract and dexamethasone.

Extract	IC_50_ (µg/mL)
APSP	ND
APE	31.140
APE-2A	> 1000
APE-2B	> 1000
APE-2C	6.080
Dexamethasone	1.000

ND, not determined.

### HPLC analysis of andrographolide and 14-deoxy-11,12-didehydroandrographolide

The calibration curves of andrographolide and 14-deoxy-11,12-didehydroandrographolide were performed at different concentrations (5, 10, 25, 50 and 100 μg/mL). The amount of each andrographolide and 14-deoxy-11,12-didehydroandrographolide was calculated based on a linear equation: Y = 57.008X + 133.85, R^2^ = 0.9999 and Y = 24.467X + 15.41, R^2^ = 0.9999, respectively. Each calibration point was conducted in triplicate. APE-2C showed the amounts of andrographolide and 14-deoxy-11,12-didehydroandrographolide at 51.68 ± 0.47%w/w and 48.22 ± 0.50%w/w, respectively while they were presented as lower than LOQ in APE-2B (Table 4).

**Table 4:** Andrographolide and 14-deoxy-11,12-didehydroandrographolide content in extracts.

Compounds	APSP (%w/w)	APE (%w/w)	APE-2B (%w/w)	APE-2C (%w/w)
Andrographolide	5.10 ± 0.18	19.58 ± 0.06	< LOQ*	51.68 ± 0.47
14-deoxy-11,12-didehydroandrographolide	< LOQ*	21.17 ± 0.15	< LOQ*	48.22 ± 0.50

*LOQ, limit of quantification; LOQ of andrographolide and 14-deoxy-11,12-didehydroandrographolide were 0.04 and 0.42 μg/mL, respectively.

### Structural characterization of chemical constituents in fraction APE-B and APE-C

Untargeted identification of metabolites in this study aimed to putatively identify the metabolites in the active fraction APE-2B and APE-2C. The total ion chromatogram (TIC) (Fig. 8). showing that the metabolites were distributed with different fraction putatively identify 37 metabolites in both fractions. This putative identification was produced from MS^2^ fragmentation that had been confirmed and compared with the literature. Those compounds consisted of metabolites from the group of terpenoids, flavonoids, phenolic, fatty acid and other groups.

#### Terpenoids

Terpenoid compounds were found in both fractions especially fraction APE-C. There were 6 compounds including andrographolide (5), parthenin (6), ginsenoside Rh5 (28), 16-hydroxy-8 (17),13-labdadien-15,16-olid-19-oic acid (35), neoandrographolide (36) and 14-deoxy-11,12-didehydroandrographolide (37).

#### Flavonoids and phenols

Four flavonoid compounds were identified in fraction APE-B including 6-Hydroxy-4′,7-dimethoxyisoflavone (13), 4H-1-Benzopyran-4-one,2-[3,4-bis(phenylmethoxy)phenyl]-3-hexyl-5,7,8-trimethoxy (29), gaudichaudiic acid G (30) and dihydrogambogic acid (34). Three phenolic compounds were also identified in fraction APE-B including 3-hydroxy-3-phenylpropanoic acid (1), 4'-hydroxyacetophenone (2) and 4-(3-hydroxybutyl)-2-methoxyphenol (4).

#### Fatty acid

Fatty acids were also identified in only fraction APE-B including 2,4-dimethylpimelic acid (3), methyl 2E,4E,6Z-decatrienoate (7), 9-hydroxy-2Z,5E,7Z,11Z,14Z-eicosapentaenoic acid (8), 8,11,14,18-eicosatetraynoic acid (11), 9-hydroxy-10-octadecen-12-ynoic acid (14), lactobionic acid (16), chloroxanthin (17), 18-hydroxylinoleic acid (18), 6Z,9Z,12Z-octadecatrienoic acid (19), 13-hydroxy-9Z,11E-octadecadienoic acid (20), 3E,9Z,12Z,15Z-octadecatetraenoic acid (21), 2-hydroxyoleic acid (24), 6Z,9Z,12Z-octadecatrienoic acid (25), hydroxyoctadecadienoic acid (26), and linolenic acid (27).

#### Other groups

In addition, there were 5 compounds which contained nitrogen and sulfur in the structure including semilepidinoside A (12), 7-hydroxy-6-methyl-8-ribityl-lumazine (15), 2-oxo-4-methylthiobutanoic acid (22), 2-oxo-8-methylthiooctanoic acid (23) and caracurine V (33).

## Discussion

*Andrographis paniculata* extract has long been used in the Thai market as a dietary supplement in several formulations, which the general public can access and use to treat common colds and viral infections. In this study, we report the viral-replication inhibitory effect of the fractions obtained from *A. paniculata* ethanol extract (APE, APE-2A, APE-2B, APE-2C, and APSP) against H3N2 and HSV-1, representing enveloped RNA and DNA viruses, respectively. Moreover, the inhibitory effect of each fraction on the production of NO which is an important mediator in the regulation of inflammation was also presented.

**Figure 7:**
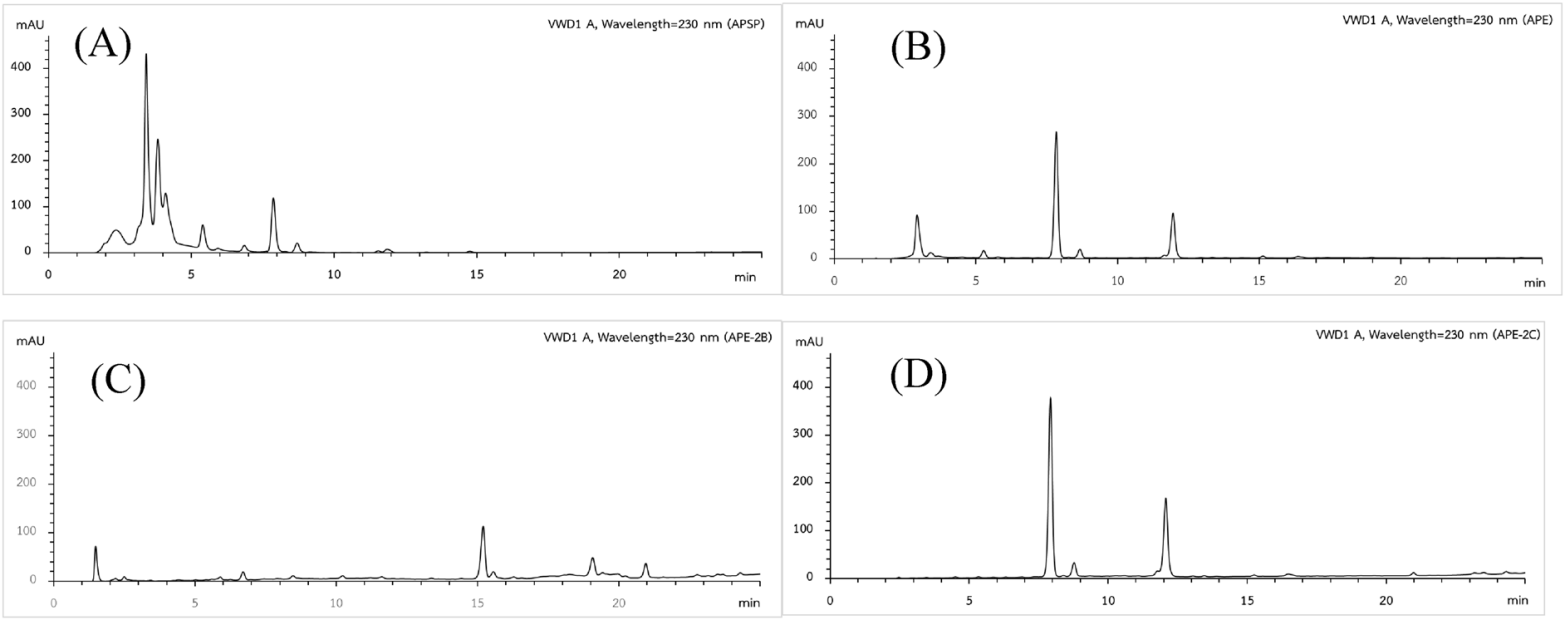
HPLC-VWD chromatogram of APSP (**A**), APE (**B**), APE-2B (**C**) and APE-2C (**D**).

**Figure 8:**
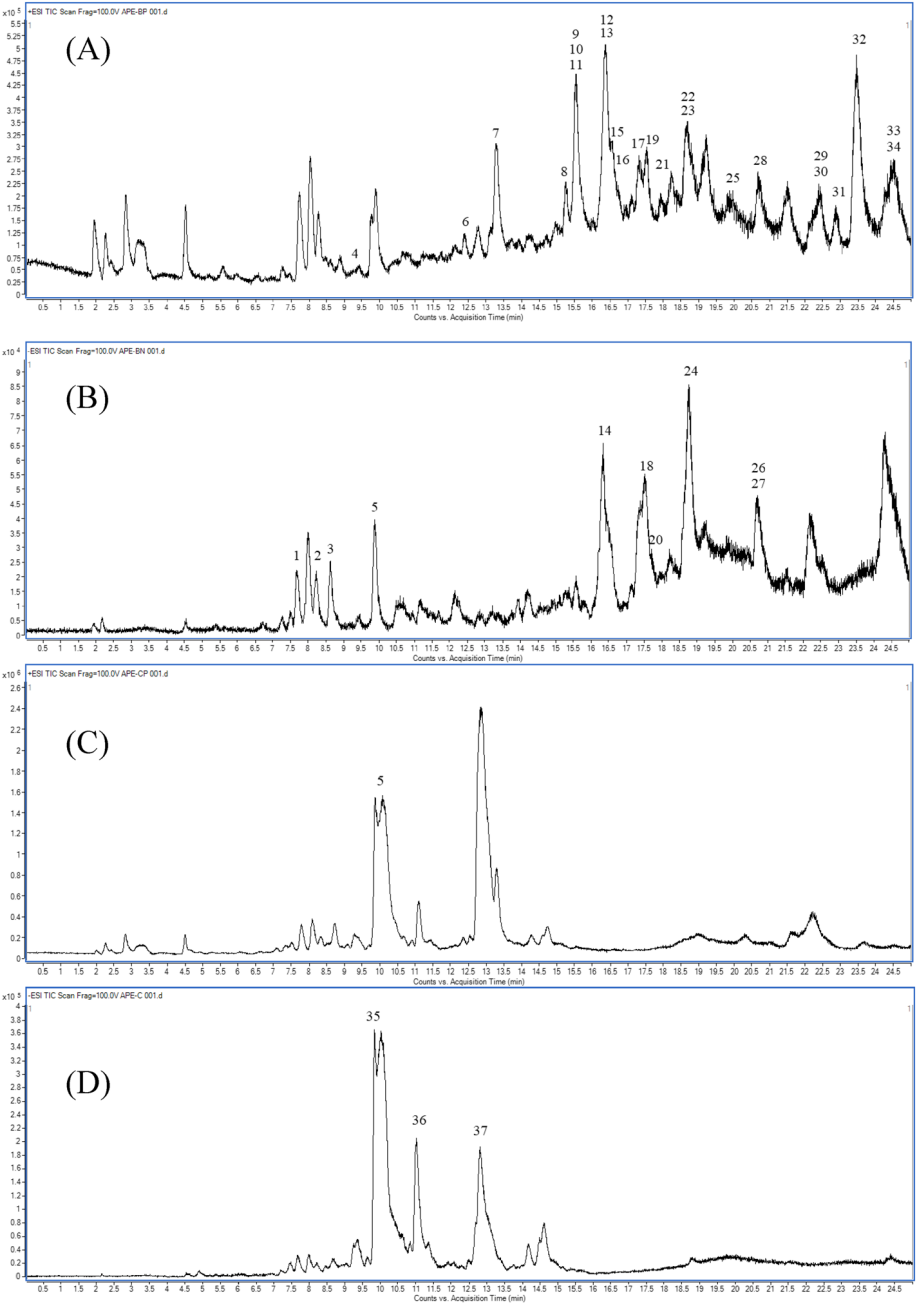
Total ions chromatogram (TIC) of fraction APE-2B in positive mode (**A**), fraction APE-2B in negative mode (**B**), fraction APE-2C in positive mode (**C**) and fraction APE-2C in negative mode (**D**). Peak numbers of compounds correspond to those in Tables 5 and 6.

The cytotoxicity of *A. paniculata* extracts to Vero and MDCK cells were different depending on concentration, reaction time and cell type. MDCK cells were more resistant to the extracts than the Vero cells. According to the results of our pre-exposure and post-exposure experiments, several *A. paniculata* extracts can directly destroy and inhibit the production of HSV-1 and influenza A (H3N2) virus particles. The APSP showed weak activity to both HSV-1 and influenza A (H3N2) with IC_50_ = 99.86 μg/mL and 275.8 μg/mL, respectively for pre-exposure experiment while IC_50_ = 114.5 μg/mL, for post-exposure experiment of influenza A (H3N2) only. This result may possible that the extraction with a high temperature of boiling and spray drying processes cause the reduction of active compounds in the extract. The degradation of andrographolide, which is the reported major component of the extract, can be occurred in the high-temperature condition^19^. The activity of APE and APE-2C on HSV-1 and influenza A (H3N2) with the IC_50_ values in the range of 7.692 μg/mL to 47.52 μg/mL (Tables 1 and 2) may be attributed to the presence of andrographolide and 14-deoxy-11,12-didehydroandrographolide shown as dominant in the extracts. HPLC analysis showed the amount of andrographolide and 14-deoxy-11,12-didehydroandrographolide in both extracts were similar proportion (Table 4). It has been hypothesized that the benefits of *A. paniculata* extract depend largely on the major components, andrographolide and its derivative which showed the effective against influenza A as well as HSV-1^20,21^. This study showed that both APE and its active component andrographolide-containing fraction (APE-2C) had a potent inhibitory effect on viral multiplication. Surprisingly, the strong activity against HSV-1 and influenza A (H3N2) with the IC_50_ = 2.43 μg/mL and 1.837 μg/mL, respectively for pre-exposure experiment and IC_50_ = 3.892 μg/mL and 7.327 μg/mL, respectively for post-exposure experiment displayed from fraction APE-2B (Tables 1 and 2). HPLC chromatogram of APE-2B (Fig. 7) did not show the signal of andrographolide and 14-deoxy-11,12-didehydroandrographolide but showed only less abundant in LC/MS analysis (Fig. 8). Fraction APE-2B has displayed several compounds with the most abundant is the fatty acid. Possible blocking mechanism of virus-entering cells; The compound blocks the cellular receptor, thus interfering with the virus-binding, or the compound blocks the virus-binding moiety (RBD of spike), thus interfering with the cellular receptor or the compound disrupts the lipid membrane of the virus, thus disintegrating the virus particle. However, this point was not conclusive yet on which compounds cause the activity in this fraction. Further studies need to be done to find the chemical markers.

**Table 5:** MS data of (+/−) ESI- QTOF-MS/MS spectra and the identification of fraction APE-2B.

Peak	RT	m/z (MS/MS)	Adduct	Formula	Tentative identification	Group
1	7.509	165.0546 (101.5941, 77.0416, 59.015)	[M−H]−	C_9_H_10_O_3_	3-Hydroxy-3-phenylpropanoic acid	Phenol
2	8.222	135.0447 (93.0345, 65.0408)	[M−H]−	C_8_H_8_O_2_	4′-Hydroxyacetophenone	Phenol
3	8.682	187.096 (169.0872, 125.0973, 97.0665)	[M−H]−	C_9_H_16_O_4_	2,4-Dimethylpimelic acid	Fatty acid
4	8.892	197.1219 (179.1105, 107.0878,105.0717, 77.0400)	[M+H]+	C_11_H_16_O_3_	4-(3-Hydroxybutyl)-2-methoxyphenol	Phenol
5	9.885	701.4418 (351.2246, 333.2136, 315.2039, 297.1914, 257.1582, 187.1524, 111.0826)	[2M+ H]+	C_20_H_30_O_5_	Andrographolide	Terpenoids
		351.2247	[M+H]+	C_20_H_30_O_5_		
		395.2004 (331.1873, 287.1980, 239.1765)	[M+HCOO]−	C_20_H_30_O_5_		
6	12.401	263.1313 (235.0967, 81.0710)	[M+H]+	C_15_H_18_O_4_	Parthenin	Terpenoids
7	13.291	181.1264 (163.1144, 135.1194, 107.0877, 77.0399)	[M+H]+	C_11_H_16_O_2_	Methyl 2E,4E,6Z-decatrienoate	Fatty acid
8	15.249	319.2338 (301.2227, 289.226, 219.1420, 205.1265, 179.1100, 153.0938, 135.1190, 123.1184, 109.1030, 95.0869, 81.0713, 67.0552, 55.0552)	[M+H]+	C_20_H_30_O_3_	9-Hydroxy-2Z,5E,7Z,11Z,14Z-Eicosapentaenoic acid	Fatty acid
9	15.436	194.1217 (179.0978, 166.0897, 151.0656, 134.0628, 107.0751, 79.0556)	[M+H]+		Unidentified	–
10	15.46	183.1418 (95.0882, 81.0714, 67.0548, 55.0552)	[M+H]+		Unidentified	–
11	15.539	301.114 (197.0487, 182.0247, 164.0139, 136.0182, 80.0273, 52.0314)	[M+H]+	C_20_H_28_O_2_	8,11,14,18-Eicosatetraynoic acid	Fatty acid
12	16.289	359.1203 (343.0905, 311.0611, 197.0496, 149.0618)	[M+Na]+	C_16_H_20_N_2_O_6_	Semilepidinoside A	Other
13	16.373	299.0981 (283.0656, 255.0707, 105.0352)	[M+H]+	C_17_H_14_O_5_	6-Hydroxy-4′,7-dimethoxyisoflavone	Flavonoids
14	16.437	293.2106 (223.1686, 183.1374, 96.9605, 59.0149)	[M−H]−	C_18_H_30_O_3_	9-Hydroxy-10-Octadecen-12-ynoic acid	Fatty acid
15	16.575	329.1092 (314.0851, 299.0608, 268.0778, 180.0086, 165.0213, 119.0508, 91.0559, 55.0184)	[M+H]+	C_12_H_16_N_4_O_7_	7-Hydroxy-6-methyl-8-ribityl lumazine	Other
16	16.724	359.1202 (344.0958, 329.0721)	[M+H]+	C_12_H_22_O_12_	Lactobionic acid	Fatty acid
17	17.313	557.4677 (187.1471, 145.1045, 133.1030, 119.0874, 105.0719, 95.0867, 81.0712, 67.0556, 55.0552)	[M+H]+	C_40_H_60_O	Chloroxanthin	Fatty acid
18	17.477	295.2292 (277.2182, 183.1398, 171.1026, 155.1079, 139.1099, 111.0811, 83.0508, 59.0125)	[M−H]−	C_18_H_32_O_3_	18-Hydroxylinoleic acid	Fatty acid
19	17.492	279.2377	[M+H]+	C_18_H_30_O_2_	6Z,9Z,12Z-Octadecatrienoic acid	Fatty acid
20	17.523	295.2298	[M−H]−	C_18_H_32_O_3_	13-Hydroxy-9Z,11E-octadecadienoic acid	Fatty acid
21	17.938	277.222	[M+H]+	C_18_H_28_O_2_	3E,9Z,12Z,15Z-Octadecatetraenoic acid	Fatty acid
22	18.78	149.0261 (121.0309, 93.0349, 65.0398)	[M+H]+	C_5_H_8_O_3_S	2-Oxo-4-methylthiobutanoic acid	Other
23	18.783	205.09 (149.0260, 121.0293, 93.0347, 65.0397)	[M+H]+	C_9_H_16_O_3_S	2-Oxo-8-methylthiooctanoic acid	Other
24	18.812	297.2543 (279.2327, 183.0126, 155.1076, 119.0491)	[M−H]−	C_18_H_34_O_3_	2-Hydroxyoleic acid	Fatty acid
25	19.21	279.2375		C_18_H_30_O_2_	6Z,9Z,12Z-Octadecatrienoic acid	Fatty acid
26	19.297	295.2393 (183.0127, 57.0344)	[M−H]−	C_18_H_32_O_3_	Hydroxyoctadecadienoic acid	Fatty acid
27	20.694	277.2188	[M−H]−	C_18_H_30_O_2_	Linolenic acid	Fatty acid
28	20.974	675.4449 (476.2812, 377.2153)	[M+Na]+	C_37_H_64_O_9_	Ginsenoside Rh5	Terpenoids
29	22.402	609.2841 (531.2503, 485.2428)	[M+H]+	C_38_H_40_O_7_	4H-1-Benzopyran-4-one,2-[3,4-bis(phenylmethoxy)phenyl]-3-hexyl-5,7,8-trimethoxy-	Flavonoids
30	22.436	625.2793 (538.2676, 465.2392)	[M+H]+	C_38_H_40_O_8_	Gaudichaudiic acid G	Flavonoids
31	22.876	609.2843 (591.2726, 559.2442, 531.2508, 485.2433)			Unidentified	–
32	23.461	593.2891 (533.2656, 505.2369, 461.2428, 433.2458)			Unidentified	–
33	24.238	607.3048 (533.2663, 461.2438)	[M+Na]+	C_38_H_40_N_4_O_2_	Caracurine V	Other
34	24.563	653.3115 (607.2668, 565.2537, 503.2540)	[M+Na]+	C_38_H_46_O_8_	Dihydrogambogic acid	Flavonoids

**Table 6:** MS data of (+/−) ESI- QTOF-MS/MS spectra and the identification of fraction APE-2C.

Peak	RT	m/z (MS/MS)	Adduct	Formula	Tentative identification	Group
35	9.632	393.1937 (329.1724, 299.1680, 285.1869, 255.1716, 137.0925)	[M+HCOO]−	C_20_H_28_O_5_	16-Hydroxy-8(17),13-labdadien-15,16-olid-19-oic acid	Terpenoids
5	9.834	395.21 (331.1929, 287.2028, 162.0675)	[M+HCOO]−	C_20_H_30_O_5_	Andrographolide	Terpenoids
		701.443 (351.2248, 333.2134, 315.2025, 297.1915, 257.1589, 227.1118, 205.1631, 187.1520)	[2M+H]+	C_20_H_30_O_5_		
36	11.012	525.2758 (479.2702, 331.1965, 161.0466, 101.0251)	[M+HCOO]−	C_26_H_40_O_8_	Neoandrographolide	Terpenoids
		503.2741	[M+Na]+			
37	12.785	333.2144 (297.1907, 273.1903, 133.1035, 119.0881, 105.0719, 95.0868, 81.0713, 67.0553)	[M+H]+	C_20_H_28_O_4_	14-Deoxy-11,12-didehydroandrographolide	Terpenoids
		331.1979 (303.2013, 255.1783, 108.0225, 57.0346)	[M−H]−			

*Andrographis paniculata* extracts were also evaluated for their efficacy in inhibiting NO production in LPS-activated RAW 264.7 macrophages. The test results showed that APE-2C, a fraction containing andrographolide, suppresses NO production with an IC_50_ of 6.08 μg/mL, whereas the APE exhibits an IC_50_ of 31.14 μg/mL. The higher activity, more than 5 times, can be attributed to the higher percentage of andrographolide in APE-2C compared to APE resulting from the fractionation process. It is important to note that the results of the anti-inflammatory activity assessments for fractions APE-2B demonstrated an absence of NO inhibitory effect primarily due to the lack of andrographolide, as confirmed through high-performance liquid chromatography (HPLC) analysis. This finding supports the anti-inflammatory activity of two *A. paniculata* fractions, which is considered beneficial against viral infections-induced inflammation.

## Conclusions

Here, we demonstrated the efficiency of *A. paniculata* extracts on their inhibition of viral multiplication using HSV-1 and influenza A (H3N2) virus, representing DNA and RNA viruses, respectively. The potent anti-viral activity together with the favorable inhibition NO production support further development of *A. paniculata* extract against virus infection. Surprisingly, the fraction with potent antiviral activity, APE-2B, lacked andrographolide and its major derivative (14-deoxy-11,12-didehydroandrographolide), indicating that other compounds in the extract may contribute to the antiviral activity. Based on our finding, it is conceivable that further exploration and development of *A. paniculata* extract may be warranted for its potential application in the development of antiviral and anti-inflammatory pulmonary products.

## Methods

### Chemicals and reagents

Acetonitrile and water (LC–MS reagent) were from JT Baker (Mallinckrodt Baker, Inc. Phillipsburg, NJ, USA). Formic acid (analytical grade) was obtained from Merck (Darmstadt, Germany). A 0.45 μm nylon disposable membrane filter and syringe filter were supplied from Vertical Chromatography (BKK, Thailand). Methanol (HPLC grade), ethanol, hexanes and ethyl acetate were supplied from RCI Labscan (BKK, Thailand). Silica gel (SiliaFlash Irregular Silica Gel P60, 40–63 µm, 60 Å) was a product from SiliCycle Inc. (Quebec, Canada). The authenticated andrographolide and 14-deoxy-11,12-didehydroandrographolide along with lipopolysaccharide (LPS), MTT [1-(4,5-dimethylthiazol-2-yl)-3,5-diphenylformazan], dexamethasone (D4902), sulphanilamide and naphthyl ethylene diamine dihydrochloride (NEDD) were purchased from Sigma–Aldrich, Inc. (St. Louis, MO, USA). Dulbecco’s modified Eagle’s medium (DMEM) and Fetal bovine serum (FBS) were supplied by Gibco Life Technologies (Grand Island, NY, USA).

### Plant materials, extraction, and isolation

The aerial part of *A. paniculata* belonging to the family Acanthaceae was collected from Phitsanulok Province, Thailand. The plant collection method and experimental use were in accordance with all the relevant guideline. The voucher specimen, collection-number 05831, was deposited at the PNU herbarium located at the Faculty of Sciences, Naresuan University. Air-dried aerial parts of *A. paniculata* (530.2 g) were extracted with 95% EtOH over a period of 3 days at room temperature and evaporated under reduced pressure to obtain a *A. paniculata* crude extract (APE, 43.08 g). The APE was further subjected to column chromatography over silica gel (SiliaFlash Irregular Silica Gel P60, 40–63 µm, 60 Å) using hexane as eluent and increasing the polarity with EtOAc (100% hexanes—100% EtOAc) to yield 3 fractions (APE-2A, APE-2B and APE-2C). The dry powder extract of *A. paniculata* (APSP) was prepared from air-dried plant sample (550.1 g) boiled with 5 L of distilled water for 30 min, then applied to mini-spray dryer (BUSHI Mini Spray Dryer B-290).

### Cell culture

Vero (ATCC, CCL-81) cells, a continuous epithelial cell line isolated from African green monkey kidney cells (*Cercopithecus aethiops*) since 1967, was used to multiply HSV-1 while Madin-Darby Canine Kidney (MDCK) (ATCC, CCL-34) cells, a continuous epithelial cell line derived from a kidney of an apparently normal adult female cocker spaniel dog (Canis familiaris) since 1958 by S.H. Madin and N.B. Darby, was used for influenza A (H3N2) multiplication. Vero cells were cultured in M199 growth medium [Medium 199 (Gibco, USA) supplemented with 10% heat-inactivated FBS (Gibco, USA), 100 units/mL of penicillin, 100 µg/mL of streptomycin, 10% sodium bicarbonate, and 1M HEPES whereas MDCK cells were cultured at 37 °C with 5% CO_2_ in MEM (Gibco, USA) growth medium. Both cells were sub-cultured every 2–3 days. When the cell monolayer was observed, the medium was discarded. Subsequently, the cell monolayer was washed with 1 × phosphate buffer saline (PBS), pH 7.4 twice. Next, pre-warmed trypsin–EDTA (0.1% for Vero cells and 0.25% for MDCK cells) was added and incubated at 37 °C for 2–3 min or until the round shape cell was observed. After that, trypsin was discarded, and the cell culture flask was gently knocked to detach the cells. The cells were then resuspended with the growth medium and sub-cultured at a split-ratio of 1:3 (Vero cells) and 1:5 (MDCK cells).

### Virus stock preparation

Standard HSV-1 strain KOS and influenza A (H3N2) viruses were used throughout the study. HSV-1 (KOS) at multiplicity of infection (MOI) of 0.01 were infected in vero cells while INF A (H3N2) at MOI of 10 were infected in MDCK cells. After inoculation, the cells were incubated for 1 h at 37 °C (rocking every 15 min) and washed once with 1xPBS, pH 7.4. Next, the M 199 maintenance medium (Medium 199 supplemented with 2% FBS, 100 units/mL of penicillin, 100 µg/mL of streptomycin, 10% sodium bicarbonate, and 1M HEPES) was added in HSV-1 infected cells. For influenza A (H3N2) infected MDCK cells, MEM maintenance medium [serum-free MEM with 1 µg/mL l-tosylamido 2-phenylethyl chloromethyl ketone (TPCK)-treated trypsin (Sigma-Aldrich, Inc., St. Louis, MO, USA)] was added. The infected cells were incubated at 37 °C for 48–72 h or until cytopathic effect (CPE) was observed. The HSV-1 infected Vero cells were frozen at − 80 °C and thawed at 37 °C for 3 times and centrifuged at 350 × g, 4 °C for 5 min whereas the influenza A (H3N2) infected MDCK cells were subsequently centrifuged without freeze–thaw process. The viruses in supernatant were aliquoted and kept at − 80 °C until used. The viral titer was determined by plaque titration assay.

### Plaque titration assay

*HSV-1 titration*: The stock seed HSV-1 was diluted (ten-fold serial dilutions) and 50 µL of the diluted HSV-1 were added to 96-well plate (Thermo Scientific, China) in quadruplicate. Next, 50 µL of Vero cells (3 × 10^4^ cells/well) were added to each well. The plate was incubated at 37 °C for 3 h. After that, 100 µL of overlay medium with 0.8% gum tragacanth in M199 growth medium were added and incubated at 37 °C for 3–4 days. Then, the plaques were developed as follows. The overlayer was removed and replaced by 100 µL of 1% crystal violet in 10% formaldehyde solution. After 45 min later, the plate was washed with running tap-water and air-dried at room temperature. The number of plaques was counted. Finally, the viral titers were calculated (plaque forming units per milliliter; PFU/mL). *Influenza A (H3N2) titration*: MDCK cells were plated at a density of 1.3 × 10^5^ cells/well in 24-well tissue culture plate (Nunc, Denmark) and incubated overnight. 100 µL of ten-fold serial dilution of influenza A viruses (H3N2) were added into each well in duplicates. The plate was incubated at 37 °C in a 5% CO_2_ incubator for 1 h (rocking gently every 15 min). After that, the viruses were removed and 0.5 mL of overlay medium (1% low melting agarose in serum-free MEM with 1 µg/mL TPCK-trypsin) were added and incubated at 37 °C for 2–3 days. Subsequently, the plaques were developed similar to previous described.

### Cytotoxicity assay

CellTiter 96® AQueous One Solution Cell Proliferation Assay (MTS) (Promega, USA), a cell metabolic assay was used. The principle is based on detection of the living cells. The living cells will use MTS tetrazolium compound to produce formazan substance. The formazan was detected by using NAD(P)H-dependent dehydrogenase enzymes. The Vero cells (3 × 10^4^ cells/well) and MDCK cells (6 × 10^4^ cells/well) were treated with various concentrations (as indicated) of herb solutions for 24 and 48 h at 37 °C. Ten microliters of MTS reagent were then added and further incubated for 4 h. Then, the mixture was measured the absorbance at 450 nm (nm) using a microplate reader (PerkinElmer, USA). The control was the untreated cells. Another control background was the medium and MTS reagent. Survival rate (%) was calculated according to instruction of the manufacturer. Three independent experiments with duplication were done.

### Plaque reduction assay (PRA)

*For pre-exposure*, various concentrations (two-fold dilution) of the herb solution were mixed with fixed amount (1 × 10^5^ PFU) of either HSV-1 or influenza A (H3N2) virus for 60 min at room temperature. After that, the viral titer was determined by plaque assay. *For post-exposure*, the virus (1 × 10^5^ PFU) was first inoculated for 60 min onto overnight cell culture of 1 × 10^5^ cells/well for Vero cells, 1.3 × 10^5^ cells/well for MDCK cells grown in 24-well plate.After that, the cells were wash once with PBS, pH 7.4 and then various concentrations (two-fold dilution) of the herb solution was added onto the cells and incubated for 24 h. The viral production was quantitated by plaque assay. 50% inhibitory concentration (IC_50_) was calculated compared to the untreated viruses. Three independent experiments with duplication were done.

### Nitric oxide (NO) inhibitory assay

The Griess reaction was used to determine the level of NO production in the medium as described in previous report^22^. Briefly, RAW 264.7 cells (1 × 10^5^ cells/well) were seeded in 96-well plates in DMEM containing 10% FBS. After incubation for 24 h, the cells were pretreated with different concentrations of the extract or vehicle (DMSO) for 2 h and then stimulated with LPS (1 μg/mL) for 18 h. The culture supernatant was collected and mixed with Griess reagent. Dexamethasone was used as a positive control. All experiments were performed in triplicate. Cell viability was performed using MTT assay. Briefly, MTT solution (0.5 mg/mL DMEM) was added to each well and then incubated for 4 h in a humidified atmosphere. After the incubation period, the supernatant was removed and DMSO was added to dissolve formazan crystals. The absorbance was measured at 570 nm using microplate reader. The results were calculated and presented as the percentage of cells viability.

### Determination of andrographolide and 14-deoxy-11,12-didehydroandrographolide in active fraction by HPLC

A stock solution containing of mixture standard compounds, andrographolide and 14-deoxy-11,12-didehydroandrographolide (1 mg/mL) in methanol, was prepared and diluted with methanol for creating a standard curve (ranging from 5.0 to 100 μg/mL). The active fraction APE-2B and APE-2C (5 mg each) were weighted and dissolved in 1 mL methanol to produce 5 mg/mL stock solutions. These stock solutions were then diluted with methanol to a concentration of 0.25 mg/mL for APE-2C and 1 mg/mL for APE-2B. The sample solutions were then filtered through a 0.45 μm nylon membrane syring filter and 20 μL of each was injected into a reverse phase Phenomenex C18 column (250 mm × 4.6 mm, 5 μm) using an Agilent 1260 infinity HPLC equipped with UV/VWD system (Singapore). The mobile phase was composed of water (A) and methanol (B) with the linear gradient program using a flow rate of 0.7 mL/min as follows: 0 to 15 min, 60–90% B; 15 to – 20 min, 90–100% B; 20 to – 25 min, 100% B and then held with 60% B for 5 min. The UV detector set at 230 nm with injection volume of 20 μL.

### Phytochemical screening by LC-ESI-QTOF-MS/MS

The LC-ESI-QTOF-MS/MS system consist of HPLC unit 1260 infinity Series (Agilent Technologies, Waldbronn, Germany) coupled with a 6540 ultra-high-definition accurate mass spectrometer (Agilent Technologies, Singapore). Chromatographic separation of the andrographolide extract (concentration 10 mg/mL) was carried out on a Luna C_18_ column (150 mm × 4.6 mm, 5 μm, Phenomenex, USA) at a flow rate of 0.5 mL/min. The column temperature was kept at 35ºC. The mobile phase consisted of a combination of A (0.1% formic acid in type I water, v/v) and B (0.1% formic acid in acetonitrile, v/v). The elution gradient from 25 to 95% B in 15 min hold on for 10 min and post run for 5 min. The injection volume was 10 μL using auto sampler. The dual electrospray ionization (ESI) source was operated in both negative and positive mode. The ESI condition was as follows: drying gas (N_2_ gas) temperature: 350 °C, gas flow rate: 10 L/min, nebulizer pressure: 30 psig, mass range: 100–1000 m/z, scan rate 4 spectra/s, capillary voltage 3500 V, skimmer voltage 65 V, octapole RFV 750 V and fragment voltage 50 V respectively. The automatic fragmentation pattern was set with collision energy at 10, 20 and 40 V using UHP N_2_ gas. Accurate mass measurements (error < 5 ppm for analytes) were obtained by means of an automated calibrant delivery system on a daily using a dual-nebulizer ESI source (calibrant solution B, Agilent Technologies, USA). Two reference masses were constantly introduced during the acquisition and used for drift correction (calibrant solution A, Agilent Technologies, USA). All acquisition and analysis of the data used MassHunter Data Acquisition Software Version B.05.01 and MassHunter Qualitative Analysis Software B 06.0 (Agilent Technologies, USA). To identify the compounds, peak retention time, mass data, and their fragmented ions were compared to those of registered compounds on public databases: Human Metabolome Database (HMBD), lipid maps, METLIN Metabolomics Database and Library (Agilent technology). The mass error was calculated when comparing a theoretical m/z and an experimentally observed m/z of an assignment.

### Supplementary Information

Supplementary Figure 1.

## Data Availability

The datasets used and/or analysed during the current study available from the corresponding author on reasonable request.
