# Supplementary material for: Inhibitory efficiency of Andrographis paniculata extract on viral multiplication and nitric oxide production
Source: Sci Rep. 2023 Nov 13;13:19738. doi: 10.1038/s41598-023-46249-y (PMC10643440; doi:10.1038/s41598-023-46249-y)
Supplement: Supplementary file 1 — Supplementary Figure 1. [file 41598_2023_46249_MOESM1_ESM.docx]

**Inhibitory efficiency of *Andrographis paniculata* extract on viral multiplication and nitric oxide production**

Ittipon Siridechakorn^1^, Parvapan Bhattarakosol^2,^*, Thanayod Sasivimolrattana^2^, Sasiprapa Anoma^2^, Eakkaluk Wongwad^3^, Nitra Nuengchamnong^4^, Ekasit Kowitdamrong^2^, Siwaporn Boonyasuppayakorn^2^, Neti Waranuch^1,5,^*

^1^Cosmetics and Natural Products Research Center, Faculty of Pharmaceutical Sciences Naresuan University, Phitsanulok, 65000, Thailand

^2^Center of Excellence in Applied Medical Virology, Department of Microbiology, Faculty of Medicine, Chulalongkorn University, Pathumwan, Bangkok, 10330, Thailand

^3^Department of Cosmetic Sciences, School of Pharmaceutical Sciences, University of Phayao, Phayao, 56000, Thailand

^4^Science Laboratory Centre, Faculty of Science, Naresuan University, Phitsanulok, 65000, Thailand.

^5^Department of Pharmaceutical Technology, Faculty of Pharmaceutical Sciences and Center of Excellence for Innovation in Chemistry, Naresuan University, Phitsanulok, 65000, Thailand

*Corresponding author: Neti Waranuch and Parvapan Bhattarakosol

Email address: [netiw@nu.ac.th](mailto:netiw@nu.ac.th), [bhparvapan@gmail.com](mailto:bhparvapan@gmail.com)

Tel: +66-55-968635, Fax: +66-55-968810

**Supplementary Figure**


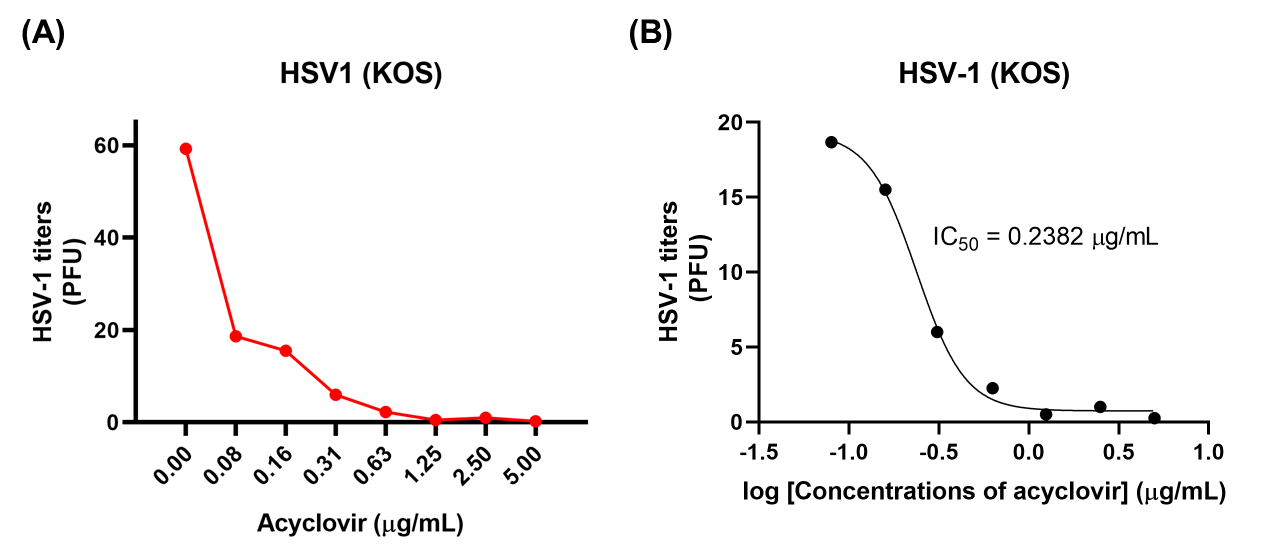


**Supplemental Figure 1.** HSV-1 susceptibility test for acyclovir treatment Thirty PFU/well of HSV-1 (KOS) were mixed with various concentrations of acyclovir (acycloguanosine,> 99% HPLC, Sigma, USA) in 0.8% gum tragacanth overlayer medium. Then, the mixture was added to Vero cells (3x10^4^ cells/well). The acyclovir susceptibility of the virus was determined by a plaque titration assay. (A) HSV titers (B) IC_50_
